# Diagnosis and Treatment of Spondylodiscitis: Insights From a Five-Year Single-Center Study

**DOI:** 10.7759/cureus.74192

**Published:** 2024-11-21

**Authors:** Diana Lima, Nuno Lopes, Ana Lúcia Pereira, Diogo Rodrigues, Marta Amaral-Silva, Elsa Marques

**Affiliations:** 1 Physical Medicine and Rehabilitation, Hospital Curry Cabral, Unidade Local de Saúde de São José, Lisbon, PRT

**Keywords:** lumbar abscess, rehabilitation, spinal cord infection, spine surgery, spondylodiscitis

## Abstract

Introduction: Spondylodiscitis is a rare but increasingly infectious disease affecting the intervertebral discs and vertebrae.

Methods: This study retrospectively analyzed 36 patients admitted with spondylodiscitis over a five-year period, examining demographics, clinical features, risk factors, causative agents, treatment approaches, and outcomes.

Results: The patient cohort had a mean age of 53.7 years, with a slight male predominance (55.6%, n=20). The most common symptoms were pain corresponding to 83.3% (n=30) followed by fever in 44.4% (n=16), and neurological symptoms were observed in 41.7% (n=15) of cases. The lumbar spine was the most frequently affected region (47.2%, n=17), and abscess formation, particularly involving the psoas muscle, occurred in 37.5% of cases. Notably, *Mycobacterium tuberculosis* was the predominant causative agent representing 50% (n=18) of all cases. All patients received antibiotic treatment, and 47.2% (n=17) required additional surgical intervention. Rehabilitation was initiated during hospitalization for 86.1% (n=31) of patients with six patients transferred to a rehabilitation yard. The overall mortality rate was 11.1% (n=3), primarily due to septic shock.

Conclusion: This study underscores the challenges of diagnosing spondylodiscitis and highlights the importance of a multidisciplinary approach to management, involving early rehabilitation to improve functional outcomes.

## Introduction

Spondylodiscitis is a rare disease whose incidence has been increasing over recent years, probably due to a greater capacity for diagnosis [1]. It is characterized by an infective inflammatory process that can affect the intervertebral discs, vertebrae and surrounding structures [2,3]. It has an incidence of 0.4 to 2.4 per 100,000 inhabitants/year and has a bimodal distribution, peaking during the first to second and fifth to sixth decades of life [4].

The most common location is the lumbar, possibly in correlation with its vascularization (derived from the inferior posterior intercostal, subcostal, iliolumbar, deep circumflex iliac, and inferior epigastric arteries) and higher mobility responsible for the “pump effect”. This fact is accentuated given that the hematogenous route is the most common route [4]. The main problem with spondylodiscitis is its late diagnosis, due to the clinical picture that is normally characterized by non-specific complaints such as uncharacteristic axial pain, fever or constitutional symptoms [5,6]. Despite the unspecified clinical findings, there is a set of risk factors to consider at the time of clinical suspicion.

According to the clinical practice guidelines of the Infectious Diseases Society of America (IDSA), there are five key elements that may assist in improving the decision-making process in patients affected by spondylodiscitis: the infective agent, segmental instability, abscess development, neurological compromise and focus of infection [7,8].

Pathogenicity and virulence of the infectious agent, systemic involvement of the disease, and whether the infection began from visceral organs (cardiac, gastrointestinal, or odontostomatological origin) are all factors directly linked to the severity of infection in a patient with spondylodiscitis. At the first stages of the illness, it is essential to isolate and accurately identify the organism causing the illness in order to determine the most effective antibiotic treatment. In Europe, the most frequent agent isolated is *Staphylococcus aureus* [3,5-7]. Segmental instability is defined as degeneration of the motion segment, which damages bone and modifies the integrity of the ligamentous structures surrounding the disc. The instability can result in pain, direct spread of pathogens and compression of neurological structures. An abscess is a pus-filled accumulation that may be situated in the canal area, exerting pressure on the neural tissues (spinal epidural abscesses), or in the paravertebral region, squeezing the lumbar plexus (psoas abscess). Neurological compression may result from instability or be associated with the persistence of epidural abscesses. The infection's emphasis is the site of the sickness, where the contaminated tissue frequently forms a capsule that is challenging to eliminate with antibiotic treatment alone. A parasite infection in this tissue may produce a significant bulk effect [7,8].

Our purpose with this study was to evaluate and describe our population in terms of pathogen agents, treatment needed, neurological deficits, rehabilitation enrollment and mortality rate.

## Materials and methods

A retrospective chart review of patients admitted to the Infectious Disease ward of Hospital Curry Cabral, in Lisbon, with the diagnosis of spondylodiscitis between July 2018 and June 2023 was carried out. Patient selection was made based on discharge diagnosis that should include "spondylodiscitis" or "spine infection". Patients without an imaging study were excluded. The following data were recorded: demographic data, comorbidities, clinical presentation, length of stay, type of spondylodiscitis, pathogenic agent, treatment adopted, need for hospitalization in Physical and Rehabilitation Medicine (PRM) ward, and functional evolution, sequelae and mortality.

Data from the selected patients were retrospectively entered into a Microsoft Excel sheet (Microsoft Corporation, Redmond, WA, USA). Categorical data are presented as numbers and percentages. A descriptive statistical methodology was applied to the data. Patients were not directly studied, and analysis was based on retrospective data collection without the identification of patients.

## Results

During the period of study, 36 patients were admitted with spondylodiscitis diagnosis (Table 1). A total of 55.6% were male, the mean age was 53.7±17.8 years (range, 22-87) and the mean hospital stay was 55.5 ± 31.4 days (range, 7-211).

**Table 1:** Baseline characteristics HCV: Hepatitis C virus; SD: Standard deviation

Characteristics	Total (n=36)
Age, mean ± SD (min-max); years	53.7 ± 17.8 (22-87)
Male, n (%)	20 (55.6)
European, n (%)	18 (50.0)
Portuguese, n (%)	16 (88.8)
Germany, n (%)	1 (5.6)
Ukraine, n (%)	1 (5.6)
African, n (%)	14 (38.9)
Guinea Bissau, n (%)	10 (71.4)
Angola, n (%)	2 (14.3)
Cape Verde, n(%)	2 (14.3)
Asian, n (%)	4 (11.1)
India, n (%)	2 (50)
Bangladesh, n (%)	1 (25)
Pakistan, n (%)	1 (25)
Time symptoms-diagnosis, n (%)	
<7 days	5 (13.9)
7-14 days	4 (11.1)
15-30 days	7 (19.4)
31-60 days	7 (19.4)
61-90 days	6 (16.7)
>90 days	7 (19.4)
Length of stay, mean ± SD (min-max); days	55.5 ± 31.4 (7-211)
HIV infection, n (%)	8 (22.2)
Diabetes mellitus, n (%)	5 (13.9)
HCV infection, n (%)	3 (5.6)
Chronic kidney disease, n (%)	4 (11.1)
Alcoholism, n (%)	2 (5.6)
Injectable drug use, n (%)	2 (5.6)
Neoplasy, n (%)	1 (2.8)
Liver transplant, n (%)	1 (2.8)
Presentation symptoms	
Fever, n (%)	16 (44.4)
Pain, n (%)	30 (83.3)
Neurological deficits, n (%)	15 (41.7)
Vertebral segments involved	
Cervical, n (%)	1 (2.8)
Dorsal, n (%)	9 (25.0)
Lumbar, n (%)	17 (47.2)
Cervical + dorsal, n (%)	1 (2.8)
Dorsal + lumbar, n (%)	8 (22.2)

A total of 22.2% of patients had a previous diagnosis of HIV infection, diabetes mellitus in 13.8% and chronic kidney disease in 11.1%. However, in 30.6% there was no risk factor or comorbidity. The most common symptoms were pain (83.3%) and fever (44.4%). Totally, 41.7% presented with neurological symptoms. In 13 cases (36.1%), it took more than two months of symptom evolution until a spondylodiscitis diagnosis was made/suspected. All the patients had a radiological exam compatible with spondylodiscitis. Although almost all cases had mild neurological impairment, there were two cases of paraplegia American Spinal Injury Association Impairment Scale (AIS) A and B. This corresponds to complete injury (AIS A) and incomplete lesion (only sensory function preserved below the level of injury, absence of motor function including S4 and S5 segments) both with poor prognosis.

The vertebral segment most frequently involved was lumbar (47.2%). Dorsal and dorso-lumbar involvement were respectively 25% and 22.2%. Only one case with cervical involvement was reported. In 24 patients, there was evidence of epidural or paravertebral abscess, 37.5% with involvement of the psoas major muscle. In 17 patients, there was another focus of infection besides the spondylodiscitis, most of the cases (n=10) were pulmonary tuberculosis.

A total of 88.9% of patients had a conclusive aetiologic diagnosis, and 16.7% had evidence of multiresistant agents. The predominant etiological agent was *Mycobacterium tuberculosis* (50.0%), succeeded by *S. aureus* (13.9%). The distribution of causal organisms is illustrated in Table 2.

**Table 2:** Etiologic agents of infectious spondylodiscitis (n=32)

	Total (n=32)
Micobacteria	
*Mycobacterium tuberculosis*, n (%)	18 (50.0)
Staphylococcus	
*S. aureus*, n (%)	1 (2.8)
Methicillin-resistant *S. aureus*, n (%)	4 (11.1)
*Klebsiella pneumoniae*, n (%)	2 (5.6)
*Brucella*, n (%)	2 (5.6)
*Serratia*, n (%)	1 (2.8)
*Salmonella*, n (%)	1 (2.8)
*Enterobacter*, n (%)	1 (2.8)
*Streptococcus*, n (%)	1 (2.8)
*Echinococcus*, n (%)	1 (2.8)

All patients received antimicrobial therapy. Monotherapy was possible in merely five (13.9%) patients. Surgical intervention was required in 17 (47.2%) of patients, 94.1% of patients with abscesses and 64.7% of the patients with neurological deficits. All the patients submitted to surgical treatment were either submitted to arthrodesis or arthrodesis and laminectomy. When an abscess was present and it was possible drainage was performed.

All patients were evaluated by a Physical and Rehabilitation physician and 31 (86.1%) had started a rehabilitation program during the hospital stay. In nine patients, an orthosis was prescribed for either lesion stabilization or pain control, only one was a cervical orthosis, and the other eight were either lumbar or thoracolumbar orthosis. Six (16.7%) patients were transferred to the rehabilitation department for a more intensive rehabilitation protocol. The evolution of these patients is summarized in Table 3.

**Table 3:** Evolution of patients admitted to rehabilitation ward (n=6) AIS: American Spinal Injury Association Impairment Scale; A: complete injury; B: sensory incomplete injury; D: motor incomplete injury; E: normal; F: female; L: lumbar level; M: male; T: thoracic level

Patient number	Sex	Age	Neurological deficit at admission	Neurological deficit at discharge	Rehabilitation length of stay (days)
1	F	33	AIS B T12	AIS B T12	45
2	F	27	AIS D L2	AIS E	11
3	F	34	AIS D L1	AIS E	40
4	F	53	AIS A T10	AIS A T10	93
5	M	56	AIS D T12	AIS D T12	34
6	M	23	AIS D L4	AIS E	17

The mortality rate was 11.1%, with three patients with a septic shock and one due to malignancy, with a fatality rate of 8.33%.

## Discussion

This study presents a comprehensive retrospective analysis of spondylodiscitis cases admitted to a hospital over a five-year period. Spondylodiscitis, an infection involving vertebral structures and intervertebral discs, remains challenging to diagnose due to its non-specific symptoms like axial pain and fever. This study provides valuable insights into epidemiology, clinical features, etiology agents and outcomes in affected patients.

This cohort of 36 patients had a mean age of approximately 54 years, with a slightly higher incidence in males (55.6%). While spondylodiscitis affects patients across age groups, its bimodal distribution is notable in both young adults and middle-aged to older adults, potentially due to age-related immunity changes and comorbid conditions. As an adult center, this bimodal distribution was not noticed in our study. Comorbidities such as HIV, diabetes mellitus, and chronic kidney disease were common, but 30.6% of patients had no risk factors, suggesting that spondylodiscitis can still affect those without predisposing conditions. Although pain was the predominant symptom, two patients experienced severe neurological impairment, including paraplegia, highlighting the potential for significant morbidity if diagnosis and treatment are delayed. The study emphasizes the difficulty in timely diagnosing spondylodiscitis, given its reliance on clinical suspicion and the non-specific nature of initial symptoms [1,7]. The time to diagnosis was significantly higher (>90 days) in a group of patients that were transferred from African centers to our center due to standard protocols, in some cases the time to diagnosis reached almost two years.

In terms of anatomical location, as described in the literature, the lumbar spine was most frequently affected, likely due to its vascular supply and mobility [8]. Most cases had abscess formation (both epidural and paravertebral), with 37.5% involving the psoas major muscle, which underscores the importance of imaging for accurate assessment. Notably, 50% of the infections were caused by *M. tuberculosis*, while *S. aureus* accounted for 13.9% of cases. This finding contrasts with existing literature that indicates *S. aureus* as a common causative agent in Europe, with tuberculosis surprisingly prevalent in this cohort [9,10]. This difference can be explained by a significant percentage of patients not being European. All patients received antibiotic treatment, with 47.2% requiring surgical intervention, primarily in cases with abscesses or neurological complications. This aligns with the IDSA guidelines that highlight the importance of considering abscess development, neurological compromise and other key factors in managing spondylodiscitis [8]. The study also reflects the complexity of managing multidrug-resistant infections, with 16.7% of cases involving multiresistant agents, underscoring the need for careful selection of antibiotics and, in some cases, combination therapy.

Rehabilitation played a critical role, with 86.1% of patients undergoing physical rehabilitation during hospitalization, which likely contributed to improved functional outcomes. Orthotic devices were used in cases needing stabilization, indicating an individual approach based on stability and pain control needs [2,4]. However, this study reported a mortality rate of 11.1%, primarily due to septic shock, which focuses on the potential severity of spondylodiscitis, particularly when diagnosis and intervention are delayed.

Our study had some limitations such as it included a pandemic period of two years without receiving other than COVID-19 patients; our protocols with African countries and the fact that our center is one of the biggest references in infectious diseases in Portugal may not reflect our country reality; another limitation is the fact that most of neurological symptoms were mild.

## Conclusions

This study indicates that while spondylodiscitis is uncommon, its prevalence may be increasing owing to enhanced diagnostic methods and sharp physician knowledge. The study's findings emphasize the importance of early diagnosis, as severe consequences including paraplegia were more probable with delayed care. Moreover, the elevated incidence of tuberculosis as a causal factor may indicate geographical or hospital-specific influences and suggest that physicians in analogous regions should uphold a heightened awareness for mycobacterial infections in instances of spinal infections.

This study clearly demonstrates the need for a multidisciplinary approach that includes infectious disease specialists, surgeons and rehabilitation professionals. Preliminary rehabilitation interventions seem to enhance functional recovery, since most patients got rehabilitative support during hospitalization, possibly aiding in the prevention of long-term disability. The study emphasizes the need for additional research on spondylodiscitis to determine effective management regimens, particularly with antibiotic therapy for resistant organisms and the scheduling of surgical operations. This investigation offers significant retrospective data; however, future prospective studies may yield deeper insights into prognostic factors, treatment efficacy and long-term results, thereby facilitating the establishment of more standardized treatment protocols.
